# Properties of Binuclear Rhodium(II) Complexes and Their Antibacterial Activity

**DOI:** 10.1155/MBD.1996.185

**Published:** 1996

**Authors:** Florian P. Pruchnik, Małgorzata Bień, Tadeusz Lachowicz

**Affiliations:** 1 Faculty of Chemistry University of Wrocław 14 Joliot-Curie Wrocław 50-383 Poland; 2 Institute of Microbiology University of Wrocław 63/77 Przybyszewskiego Wrocław Poland

## Abstract

Binuclear rhodium(II) complexes [Rh_2_Cl_2_(μ-OOCR)_2_(N-N)_2_], [Rh_2_(μ-OOCR)_2_(N-N)_2_(H_2_O)_2_](RCOO)_2_ and [Rh_2_Cl_2_(μ-OOCCH_3_)(terpy)_2_](H_3_O)Cl_2_.9H_2_O (R = H, Me, Bu^n^, ph, PhCHOH; N-N = 2,2′-bipyridine (bpy), 1,10-phenanthroline (phen), 2,9-dimethyl-1,10-phenanthroline (dmp) and 6,7-dimethyl-2,3- di(2-pyridyl)quinoxaline (dmpq); terpy 2,2′:6′,2′′-terpyridine) have been synthesized and their structure and properties have been studied by electronic, IR and ^1^H NMR spectroscopy. Antibacterial activity of these complexes against *Staphylococcus aureus* and *Escherichia coli* has been investigated. The most active antibacterial agents against *S. aureus* were [Rh_2_(OOCPh)_2_(phen)_2_(H_2_O)_2_]^2+^, [Rh_2_(OOCPh)_2_(dmpq)_2_(H_2_O)_2_]^2+^, [Rh_2_(OOCBu)_2_(phen)_2_(H_2_O)_2_]^2+^ and [Rh_2_-(OOCBu)_2_(bpy)_2_(H_2_O)_2_]^2+^ which were considerably more active than the appropriate nitrogen ligands. The complexes show rather low activity against *E. coli.*


### PROPERTIES OF BINUCLEAR RHODIUM(II) COMPLEXES AND THEIR ANTIBACTERIAL ACTIVITY

Florian P. Pruchnik[1], Małgorzata Bień[2], and Tadeusz Lachowicz[2]

[1] Faculty of Chemistry, University of Wrocław, 14 Joliot-Curie, 50-383 Wrocław, Poland

[2] Institute of Microbiology, University of Wrocław, 63/77 Przybyszewskiego, Wrocław, Poland

### Abstract

Binuclear rhodium(II) complexes $[Rh_2Cl_2(\mu\text{-}OOCR)_2(N\text{-}N)_2]$, $[Rh_2(\mu\text{-}OOCR)_2(N\text{-}N)_2(H_2O)_2](RCOO)_2$ and $[Rh_2Cl_2(\mu\text{-}OOCCH_3)(terpy)_2](H_3O)Cl_2{\cdot}9H_2O$ (R = H, Me, $Bu^n$, Ph, PhCHOH; N-N = 2,2'-bipyridine (bpy), 1,10-phenanthroline (phen), 2,9-dimethyl-1,10-phenanthroline (dmp) and 6,7-dimethyl-2,3-di(2-pyridyl)quinoxaline (dmpq); terpy = 2,2':6',2"-terpyridine) have been synthesized and their structure and properties have been studied by electronic, IR and $^1H$ NMR spectroscopy. Antibacterial activity of these complexes against *Staphylococcus aureus* and *Escherichia coli* has been investigated. The most active antibacterial agents against *S. aureus* were $[Rh_2(OOCPh)_2(phen)_2(H_2O)_2]^{2+}$, $[Rh_2(OOCPh)_2(dmpq)_2(H_2O)_2]^{2+}$, $[Rh_2(OOCBu)_2(phen)_2(H_2O)_2]^{2+}$ and $[Rh_2\text{-}(OOCBu)_2(bpy)_2(H_2O)_2]^{2+}$ which were considerably more active than the appropriate nitrogen ligands. The complexes show rather low activity against *E. coli*.

### Introduction

Binuclear rhodium(II) tetracarboxylato complexes $[Rh_2(OOCR)_4L_2]$ as well as complexes containing two bridging ligands $[Rh_2X_2(OOCR)_2(N\text{-}N)_2]$ and $[Rh_2(OOCR)_2(N\text{-}N)_2L_2]^{2+}$, where L = Lewis base, X = halide and N-N = 2,2'-bipyridine, 1,10-phenanthroline and their derivatives have aroused considerable interest because of interesting structure and reactivity [1-3], catalytic properties [1,4] and anticancer activity [1,2,5]. We have prepared and investigated the structure, reactivity, catalytic activity and cytostatic activity of $[Rh_2X_2(OOCR)_2(N\text{-}N)_2]$ and $[Rh_2(OOCR)_2(N\text{-}N)_2(H_2O)_2]^{2+}$ complexes (R = H, Me, PhCHOH, N-N = 2,2'-bipyridine, 1,10-phenanthroline and their derivatives [2,4]. Rhodium(II) carboxylato complexes belong to the most promising platinum metals compounds having anti-cancer and biological activity [5-7]. These compounds were shown to increase the lifespan of tumor bearing mice and in favorable cases to produce complete regression of the tumor [8-11]. This discovery has prompted investigations into the chemical properties and biological effects of these complexes [1,5,6,10-15]. The antitumor activity of $Rh_2(OOCR)_4$ complexes intensified studies of their interaction with biomolecules, e.g. aminoacids, peptides, vitamins, nucleic acid bases, adenosine phosphates, DNA, etc. [1,5,6,8-15]. Tetraacetatodirhodium(II) binds effectively to human serum albumin via imidazole rings leading to the conformational changes of HSA [15]. The binuclear rhodium(II) carboxylates, like cis-$[PtCl_2(NH_3)_2]$, appear to increase biological activity induced by irradiation [16-17].

Recently it has been found [18] that $[Rh_2(OOCR)_2(N\text{-}N)_2(H_2O)_2]^{2+}$ complexes show cytostatic activity for human oral carcinoma KB cell line *in vitro*. Some of the determined biological activities were in close relation with findings obtained on plant cell cultures [19], when synchronously cultivated green algae were exposed to Rh(II) complexes. New metal compounds possessing antimicrobial activity are recently intensively investigated because of the increasing resistance of microbes against many antibiotics. It has been found that some platinum complexes possessing antitumor activity show also antibacterial properties. A further well defined series of metal complexes with antibacterial activity is that of rhodium(III) coordination compounds of formula *trans*-$[RhX_2(py)_4]Y$ [20-22]. These complexes are more active against Gram-positive microorganisms than Gram-negative ones. However, the complexes $[RhCl_2(bpy)_2]^+$ are not active antibacterial agents. There is considerable interest in interactions of tetra-μ-carboxylato-dirhodium(II) complexes with nucleic acid bases because they function as antitumor agents against many types of tumors by inhibiting DNA or protein synthesis. The examination of the biological activity of these compounds in such living systems (i.e. tumor bearing animals) is time consuming and expensive and should be used at the last step of examination. The exposure of mammalian cells in cultures or microorganisms cultivated synchronously, or an examination of the antibacterial activity of metal compounds [19,23] give also interesting information about the general biological activity of the tested compounds. In this paper, we report on synthesis and properties of dimeric rhodium(II) complexes with carboxylato and heterocyclic nitrogen ligands and their antibacterial activity.

### Experimental

*Synthesis of the complexes.*

Complexes $[Rh_2(OOCCH_3)_4(H_2O)_2]$ [24], $Na_4[Rh_2(CO_3)_4]\cdot 2.5H_2O$ [25], $[Rh_2$-{OOCC-H(OH)Ph}$_4(H_2O)_2]$ [2], $[Rh_2${OOCCH(OH)Ph}$_2(phen)_2(H_2O)_2](OOC$-CH(OH)Ph)$_2$ (**2**) [18], $[Rh_2Cl_2${OOCCH(OH)Ph}$_2(phen)_2]$ (**3**) [2], $[Rh_2(OOCH)_2(bpy)_2Cl_2]\cdot 4H_2O$ (**8**) [2], and $[Rh_2(OOCCH_3)(terpy)_2Cl_2][H_3O]Cl_2\cdot 9H_2O$ (**9**) [26] were prepared by literature methods.

1,10-Phenanthroline (phen), 2,9-dimethyl-1,10-phenanthroline (dmp), 2,2'-bipyridine (bpy), 2,2':6',2"-terpyridine (terpy) and 6,7-dimethyl-2,3-bis(2-pyridyl)quinoxaline (dmpq) were obtained from Aldrich and used without further purification.

**[Rh$_2$(OOCCHOHPh)$_2$(dmp)$_2$(H$_2$O)$_2$](OOCCHOHPh)$_2$ (1).** A solution of $[Rh_2(OOCHOHPh)_4(H_2O)_2]$ (0.1269 g, 0.15 mmol) and dmp (0.0625 g, 0.3 mmol) in 6 cm$^3$ of ethanol was heated at reflux with stirring for 2 h. The initial green colour of the solution was soon replaced by brown-red and then by deep green-blue. The deep green-blue rhodium (I) complexes are formed because rhodium(II) dimers are reduced by ethanol. However, rhodium(I) compounds are very readily oxidized to red-brown dimeric rhodium(II) complexes under an atmosphere of air. After cooling in air atmosphere, the brown-red solution was filtered and the filtrate was treated with diethyl ether to produce a brown solid.The sample was collected by filtration in air, washed with diethyl ether, and dried in vacuo; yield 0.104 g, 55%. Anal . Calc. for $C_{60}H_{56}N_4O_{14}Rh_2$: C, 57.06; H, 4.47; N, 4.44. Found: C, 56.45; H, 3.91: N, 3.97.

**[Rh$_2$(OOCCH$_3$)$_2$(dmpq)$_2$(H$_2$O)$_2$](OOCCH$_3$)$_2$•2H$_2$O (4).** A solution of $[Rh_2(OOCCH_3)_4(H_2O)_2]$ (0.0956 g, 0.2 mmol) and dmpq (0.125 g, 0.4 mmol) in 7 cm$^3$ of ethanol was heated under reflux for 5 h, cooled to the room temperature and, after filtration in air, concentrated to approximately 2 cm$^3$.The product was deposited with diethyl ether. The dark brown precipitate was filtered off, washed with diethyl ether and dried in vacuo, yield .0.1456 g, 64 %. Anal . Calc. for $C_{48}H_{52}N_8O_{12}Rh_2$: C, 50,63; H, 4.60; N, 9.84. Found: C, 49.96; H, 4.37: N, 9.54.

**[Rh$_2$(OOCPh)$_2$(phen)$_2$(H$_2$O)$_2$](OOCPh)$_2$ (5).** A mixture of $[Rh_2$-$(OOCPh)_4(H_2O)_2]$ (0.1089 g, 0.15 mmol) and phen (0.0594 g, 0.3 mmol) in ethanol (5cm$^3$) was heated under reflux for 3 h and filtrated under air atmosphere. Addition of diethyl ether to the reaction solution produces a brown-red precipitate. The product was washed with diethyl ether and dried in vacuo, yield 0.1674 g, 77%. Anal . Calc. for $C_{52}H_{40}N_4O_{10}Rh_2$: C, 57.47; H, 3.71; N, 5.16. Found: C,56.91; H, 4.14: N, 5.33.

**[Rh$_2$(OOCCH$_3$)$_2${OOCCH(OH)Ph}$_2$] (6)** A solution of $[Rh_2(OOC$-$CH_3)_4(H_2O)_2]$ (0.221 g, 0.5 mmol) and (*S*)-mandelic acid (0.152 g, 1 mmol) was heated under reflux for 24 h. The solution was evaporated and green compound was dried in vacuo at 100 °C for 5 h, yield 0.251 g, 80 %. Anal . Calc. for $C_{20}H_{20}O_{10}Rh_2$: C, 38.36; H, 3.22; Found: C,38.00; H, 3.39.

**[Rh$_2$(H$_2$PO$_4$)$_4$(H$_2$O)$_2$] (7).** A mixture of $Na_4[Rh_2(CO_3)_4]\cdot 2.5H_2O$ (0.0583 g, 0.1 mmol) and 0.5 cm$^3$ of 1 M $H_3PO_4$ in water (5 cm$^3$) was heated for 1 h, concentrated to approximately half volume. The green powder was collected, washed with methanol and dried in vacuo, yield 0.0481 g, 67%. Anal. Calc.: Rh, 32.68; P, 19.67. Found: Rh, 31.8; P, 19.9.

**[Rh$_2$(OOCPr)$_2$(bpy)$_2$(H$_2$O)$_2$](OOCPr)$_2$ (10)** A mixture of $[Rh_2(OOCPr)_4]$ (0.111 g, 0.2 mmol) and bpy (0.0624 g, 0.4 mmol) in 1,4-dioxane (5 cm$^3$) was heated under reflux for 2 h. The red-brown precipitate was collected, washed with dioxane and dried in vacuo, yield 0.123 g, 68 %. Anal. Calc. for $C_{36}H_{48}N_4O_{10}Rh_2$: C, 47.91; H, 5.36; N, 6.21. Found: C, 47.54, H, 5.12, N, 5.88.

**[Rh$_2$(OOCBu$^n$)$_2$(phen)$_2$(H$_2$O)$_2$](OOCBu$^n$)$_2$ (11)** The procedure was analogous to that used for the previous preparation . Starting with 0.122 g (0.2 mmol) of $[Rh_2(OOCBu^n)_4]$ and 0.0792 g (0.4 mmol) of 1,10-phenanthroline hydrate, red-brown complex **11** was obtained (0.143 g, 71 % yield). Anal. Calc. for $C_{44}H_{56}N_4O_{10}Rh_2$: C, 52.49; H, 5.61; N, 5.57. Found: C, 52.82; H, 5.40; N, 5.69.

**[Rh$_2$(OOCBu$^n$)$_2$(bpy)$_2$(H$_2$O)$_2$](OOCBu$^n$)$_2$ (12)** This complex was obtained according to the procedure used for the preparation of complex **10**, starting with 0.122 g (0.2 mmol) of $[Rh_2(OOCBu^n)_4]$ and 0.0624 g (0.4 mmol) of bpy. Yield 0.146 g, 76 %. Anal. Calc. for $C_{40}H_{56}N_4O_{10}Rh_2$: C, 50.11; H, 5.89; N, 5.84. Found: C, 50.35; H, 5.45; N, 5.95.

**[Rh$_2${OOCCH(OH)Ph}$_2$(bpy)$_2$(H$_2$O)$_2$](OOCCH(OH)Ph)$_2$ (13).** A solution of $[Rh_2${OOCCH(OH)Ph}$_4(H_2O)_2]$ (0.423 g, 0.5 mmol) and 2,2'-bipyridine (0.156 g 1 mmol) in 5 cm$^3$ of ethanol was heated at reflux for 2 h, cooled to the room temperature and filtered in air. From the filtrate the dark red product was deposited by means of diethyl ether. Precipitate was filtered off, washed with $(C_2H_5)_2O$ and dried under reduced pressure, yield 0.376 g, 65%. Anal. Calc. for $C_{52}H_{48}N_4O_{14}Rh_2$: C 53.90, H 4.18, N 4.83. Found: C 53.40, H 4.07, N 4.94.

**[Rh$_2$(OOCPr)$_4$] (14).** A mixture of $Na_4[Rh_2(CO_3)_4]\cdot 2.5H_2O$ (0.583 g, 1 mmol) and $CH_3CH_2CH_2COOH$ (0.73 cm$^3$, 8 mmol) in water (10 cm$^3$) was heated for 2 h. After cooling the green precipitate was filtered off, washed with water and dried in vacuo, yield 0.458 g, 92 %. Anal. Calc for $C_{16}H_{28}O_8Rh_2$: C, 34.68; H, 5.09. Found: C, 34.28; H, 4.92.

**[$Rh_2(OOCBu^n)_4$] (15).** The procedure was analogous to that used for the previous preparation . Starting with 0.583 g (1 mmol) of $Na_4[Rh_2(CO_3)_4]\cdot 2.5H_2O$ and 0.87 cm$^3$ of $C_4H_9COOH$, green precipitate (0.555 g, 91 % yield) of tetra-μ-pentanoato-dirhodium(II) was obtained. Anal. Calc for $C_{20}H_{36}O_8Rh_2$: C, 39.36; H, 5.95. Found: C, 38.95; H, 5.86.

**[$Rh_2(OOCPh)_4(H_2O)_2$] (16).** A mixture of $Na_4[Rh_2(CO_3)_4]\cdot 2.5H_2O$ (0.583 g, 1 mmol) and PhCOOH (0.977 g, 8 mmol) in 15 cm$^3$ of water was heated for 1 h. The yellow-green precipitate was collected, washed with hot water ( 5 x 15 cm$^3$) and dried in vacuo. Anal . Calc. for $C_{28}H_{24}O_{10}Rh_2$: C, 46.30; H, 3.33; Found: C,46.37; H, 3.08.

*Physical measurement.*

Infrared spectra (KBr pellets and nujol mulls) were measured on a Perkin Elmer 283 and a Bruker IFS 113v, UV-VIS on a Cary 5 and Beckman DU 7500, and $^1$H NMR spectra on a Bruker AMX 300.

*Antibacterial activity.*

The antibacterial activity was evaluated *in vitro* according to the standard methods [27] on reference strains *Staphylococcus aureus* 209P (Oxford) and clinical isolates of *S. aureus* obtained from Bacteriological Laboratory of Kaminski's Hospital.The preliminary experiments were performed on overnight broth culture of the bacteria. Minimal inhibitory concentration (MIC) was determined by plating 0.1 cm$^3$ of broth cultures, suitably diluted in saline, on nutrient agar plates supplemented with tested compounds of different concentration. The cultures were diluted to obtain the samples spread on the control plates (without rhodium compound) containing about 100 colony forming units. Activity of the compounds was also tested in liquid YP medium (yeast axtract Difco 1%, pepton Bacto 1%, NaCl 0.5%). In these tests the media containing rhodium complexes were inoculated with 10$^3$ or 10$^6$ colony forming units and viable count was determined at suitable intervals. The turbidity at 560 nm was determined after 24 h and 48 h. Survival of the bacteria was also tested in saline containing different concentration of the compounds.

### Results and Discussion

The $[Rh_2(RCOO)_2(N\text{-}N)_2(H_2O)_2][RCOO]_2$ complexes were prepared by reactions between $[Rh_2(RCOO)_4(H_2O)_2]$ and appropriate nitrogen ligand.

They are stable under air atmosphere both in solid state and in solutions, readily soluble in water, methanol and ethanol and slightly soluble in higher alcohols, insoluble in diethyl ether and nonpolar solvents. Primary and secondary alcohols at elevated temperatures reduce these complexes giving rhodium(I) complexes, that are very readily reoxidized to rhodium(II) compounds by means of air.

All complexes have been characterized by a combination of elemental analysis and IR, UV-VIS and $^1$H NMR spectroscopy. The data are collected in Tables 1-3.

The physicochemical measurements indicate that structure of the cationic complexes containing two bridging carboxylato ligands is analogous to that of $Rh_2Cl_2(RCOO)_2(N\text{-}N)_2$ [2,4] (Fig. 1) with water molecules coordinating along Rh-Rh axis.

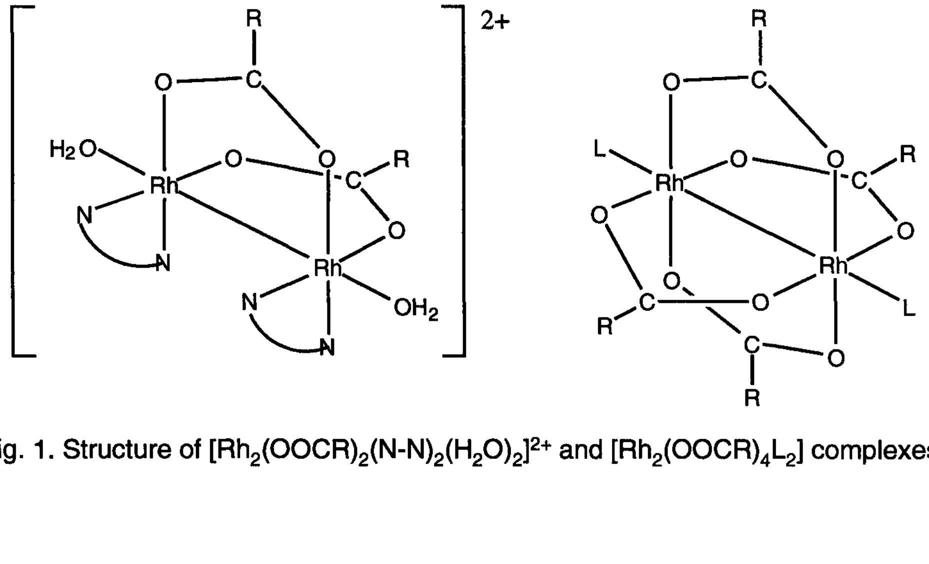

Fig. 1. Structure of $[Rh_2(OOCR)_2(N\text{-}N)_2(H_2O)_2]^{2+}$ and $[Rh_2(OOCR)_4L_2]$ complexes.

Table 1. Electronic Spectra of Rhodium(II) Complexes.

| Complex* | Band, λ(ε), [nm](M$^{-1}$cm$^{-1}$) |
|---|---|
| 1 | 560(450)sh, 460(2100)sh, 361(6300)sh, 272(34300), 225(67700) |
| 4 | 588(900)sh, 475(2800)sh,378(14400), 278(42300) |
| 5 | 559(160), 411(1600)sh, 363(2800)sh, 334(4400)sh, 271(29600), 250(48400)sh, 231{68000) |
| 6 | 587(210), 451(100),415(75)sh 340(150)sh, 252(5600), 218(20300) |
| 7 | 666(120), 467(70)sh, 400(95), 290(470)sh, 246(16300) |
| 10 | 563(240), 415(2000), 360(3400)sh, 305(15300), 272(27200)sh, 257(30500) |
| 11 | 568(370), 411(2450)sh, 332(5500), 272(32200), 254(36800), 228(45800) |
| 12 | 559(290), 415(2350), 360(4100)sh, 301(17900)sh, 271(31800)sh, |
| 13 | 565(230), 412(2100), 355(3200)sh, 312(16600)sh, 302(18800), 253(32800) |

* solutions in $H_2O$

In the electronic spectra (Table 1) of these complexes in the visible region two bands are observed. Band I occurs at about 560-568 nm, about 20-30 nm lower compared with that for $[Rh_2(RCOO)_4(H_2O)_2$ compounds. This shift may be explained by assuming that band I corresponds to the $\pi^*(Rh_2)\rightarrow\sigma^*(Rh_2)$ transition, since the $\pi^*(Rh_2)$ levels in $[Rh_2(RCOO)_2(N\text{-}N)_2(H_2O)_2]^{2+}$ complexes, due to the interaction of the orbitals of the $Rh_2$ core with $\pi^*$ orbitals of nitrogen ligands, have lower energy than that of the $\pi^*(Rh_2)(5e_g)$ orbitals in $[Rh_2(OOCR)_4(H_2O)_2]$ compounds. The intense band II at about 410-470 nm was assigned to the allowed charge-transfer transition $\sigma(Rh_2)\rightarrow\pi^*(N\text{-}N)$ [28]. This band obscures the absorption of low intensity attributed to the $\pi^*(Rh_2)\rightarrow\sigma^*(Rh\text{-}O)$ transition observed at 430-450 nm in $[Rh_2(OOCR)_4(H_2O)_2]$ complexes. The energy of the band I strongly depends on the nature of axial ligands and increases with increasing of the field strength of these ligands. Band I remains relatively constant for axial oxygen donor ligands, but is blue shifted with nitrogen or sulfur donor ligand. Electronic spectra of $[Rh_2(RCOO)_2(N\text{-}N)_2(H_2O)_2](RCOO)_2$ in ethanol in the presence of adenine for molar ratios $Rh_2$:adenine of 1:1 and 1:2 show that band I is blue shifted for about 10-40 nm. This indicate that adenine is coordinating to rhodium via nitrogen atom. However, in the case of adenosine blue shift is much lower (ca. 2-5 nm) suggesting that this ligand is coordinating through oxygen atom or undergoes stacking owing to the π interaction between the purine moiety and a heteroaromatic amine bound with rhodium atom. The stacking of nucleobases and metal complexes is well known [29-31].

Table 2. IR Spectra of Rhodium(II) Compounds.

| Complex | $\nu_{CH}$ [cm$^{-1}$] | $\nu^{as}_{COO}$ [cm$^{-1}$] | $\nu^{s}_{COO}$ [cm$^{-1}$] | other bands [cm$^{-1}$] |
|---|---|---|---|---|
| 1 | 3080vw, 3055m, 3025m, 3000vw, 2970vw, 2915vw, 2840vw | 1588vs, 1575sh, 1550sh | 1405sh, 1393vs, 1373s, 1350vs | 1527vw, 1498wm, 1480m, 1440m, 1292vw, 1285vw, 1232vw, 1210w, 1200vw, 1180m, 1160sh, 1138mw, 1100sh, 1078m, 1052s, 1018mw, 988vw, 973vw, 947m, 918w, 854s, 790m, 745s, 735s, 700s, 667mw, 637m, 560w, 545m, 470vw, 435w, 392wm |
| 2 | 3090w, 3062w, 3027w | 1610vs, 1575vs | 1428vs, 1410vs, 1400sh, 1360sh, 1338vs, 1325sh | 1513m, 1489m, 1448s, 1220w, 1205vw, 1187m, 1143w, 1110sh, 1087m, 1053s, 1023m, 997vw, 948vw, 928m, 898vw, 880vw, 840s, 782w, 735vs, 711vs, 696vs, 653vw, 644w, 612w, 558vw, 510vw, 470vw, 433vw |
| 4 | 3126, 3672vw, 2978w, 2925w, 2858w | 1571vs | 1445vs, 1412vs | 1624m, 1477s, 1362s, 1329w, 1287w, 1215w, 1187w, 1164w, 1089m, 1051w, 999m, 875m, 801w, 776m, 756m, 707m, 654w, 631vw, 619vw, 573w, 388vw, 365w, 351vw, 333w |

| | | | | |
|---|---|---|---|---|
| 5 | 3080vw, 3060w, 3040vw, 3015vw, 2920vw, 2855vw | 1592vs, 1542vs | 1427s, 1410vs, 1355sh, 1340vs | 1512m, 1492w, 1380w, 1297vw, 1220vw, 1204vw, 1168w, 1142w, 1124vw, 1108vw, 1064w, 1020m, 850m, 838s, 810vw, 766vw, 720vs, 710vs, 692s, 668m, 612vw, 502m, 433vw, 400vw, 385vw, 357vw, 327vw |
| 6 | 3090vw, 3065w, 3047vw, 3035w, 3010vw, 2930w, 2860vw | 1592vs, 1580vs | 1450sh, 1435sh, 1413vs | 1645w, 1522w, 1490w, 1370w, 1349w, 1280w, 1227w, 1184m, 1082m, 1060s, 1045sh, 1023m, 998vw, 945w, 918vw, 850w, 837vw, 782w, 750m, 694vs, 633m, 593vw, 561w, 485w, 376m, 337m |
| 9 | 3105vw, 3075wm, 3038w, 2990vw, 2925w, 2855 vw | 1545vs | 1447vs | 1601s, 1568vw, 1472s1393s, 1348w, 1330vw, 1307m, 1287m, 1270vw, 1245m, 1181w, 1158m, 1135mw, 1113vw, 1090mw, 1048mw,1023m, 972vw, 920vw, 897vw, 830vw, 817vw, 765vs, 727w, 698mw, 679w, 655w, 645wm, 618vw, 512wm, 437vw |
| 10 | 311ovw, 3078w, 3050w, 3020vw, 2960s, 2935w, 2885 | 1605m, 1580sh, 1562vs, 1557vs | 1462s, 1447s, 1430, 1418vs, 1377s | 1490vw, 1330vw, 1312m, 1290sh, 1243m, 1212vw, 1165vw, 1158vw, 1095w, 890w, 796m, 760vs, 719m, 666vw, 643vw, 418w, 360vw, 350vw |
| 11 | 3058w, 2967s, 2940s, 2880m, 2870m, 2090vw | 1590vs, 1558 | 1455sh, 1431vs, 1420vs, 1370s | 1514m, 1493w, 1305m, 1288m, 1225m, 1204m, 1145w, 1108w, 1093w, 848sh, 842vs, 776w, 763m, 714vs, 695vw, 667w, 620vw, 590vw, 552vw, 515vw, 440vw |
| 12 | 3120vw, 3090vw, 3077w, 3043w, 2970s, 2944s, 2880 | 1605sh, 1587vs, 1565vs | 1470s, 1453s, 1431vs, 1420 | 1497vw, 1379s, 1348s, 1310m, 1283w, 1250vw, 1230m, 1207vw, 1167w, 1110w, 1100vw, 1075vw, 1063vw, 1048vw, 933vw, 885vw, 809w, 772vs, 756sh, 723s, 680vw, 672vw, 610w, 533vw, 427m |
| 13 | 3117w, 3085w, 3057w, 3030w, 2927vw | 1620sh, 1605vs, 1574vs | 1420sh, 1404vs, 1335vs, 1327vs, 1310sh | 1490m, 1465s, 1448vs, 1241w, 1230vw, 1186m, 1167w, 1155w, 1082m, 1056s, 1023m, 998w, 930w, 897vw, 854vw, 760vs, 733vs, 718vs, 695vs, 670w, 642vw, 613vw, 537w, 468vw, 418w |
| 14 | 2985s, 2935m, 2877m | 1570vs | 1460s, 1427vs, 1410vs, 1380w | 1523m, 1347w, 1313s, 1262m, 1212w, 1102, 1045vw, 895w, 868vw, 798m, 744m, 701vw, 658m, 456m, 440w, 367w, 353m |
| 15 | 2983s, 2940s, 2880m | 1570vs | 1446m, 1430s, 1413vs | 1524w, 1508vw, 1469w, 1458vw, 1385w, 13622?, 1318w, 1297w, 1245vw, 1230vw, 1202w, 1109m, 762w, 735w, 682m, 667vw, 460vw, 426vw |
| 16 | 3100vw, 3080vw, 3060w, 2920w, 2850w | 1552vs | 1398vs | 1594s, 1495m, 1445m, 1310vw, 1285vw, 1260vw, 1173m, 1140m, 1069w, 1023m, 997vw, 927w, 840m, 802vw, 722s, 708s, 689s, 520sh, 512m, 482m, 462w |

The IR spectra of complexes with phenanthrolines showed that the carboxylates are symmetrically bonded to the both rhodium atoms because differences between $v^{as}$(COO) and $v^{s}$(COO) are small (Table 2).

In the $^1$H NMR spectra, chemical shifts of protons of carboxylato ligands in $[Rh_2(RCOO)_2(N\text{-}N)_2(H_2O)_2]^{2+}$ are higher than those of non-coordinated carboxylates and RCOO groups in $Rh_2(OOCR)_4(H_2O)_2$ complexes.

The phenanthroline, 2,2'-bipyridine and 2,2':6',2''-terpyridine ligands in $[Rh_2(OOCPh)_2(phen)_2(H_2O)_2](OOCPh)_2$, $[Rh_2Cl_2(OOCR)_2(N\text{-}N)_2]$ (R = H, alkyl) $[Rh_2(OOCPr)_2(bpy)_2(H_2O)_2](OOCPr)_2$, $[Rh_2(OOCBu^n)_2(phen)_2(H_2O)_2](OOCBu^n)_2$ $[Rh_2(OOCBu^n)_2(bpy)_2(H_2O)_2](OOCBu^n)_2$ and $[Rh_2(OOCCH_3)(terpy)_2Cl_2][H_3O]Cl_2{\cdot}9H_2O$ are symmetrically bound to the rhodium atoms. This is proven by the presence only 4 signals of hydrogen atoms of heterocyclic nitrogen ligands in the $^1$H NMR spectra of phenanthroline complexes and signals of H5 + H5'', H6 + H6'' and multiplet for the other protons of 2,2':6',2"- terpyridine ligand in complex **9** (Table 3). The $^{1}$H NMR spectrum of terpy complex is consistent with the X-ray structure of this compound [26]. However, the $^{1}$H NMR spectra of complexes **1-4**, and **13** consist of multiplets due to non-equivalent protons of the nitrogen ligands. Thus, the nitrogen ligands are coordinated asymmetrically in complexes containing chiral mandelato bridging groups and in acetato complex with dmpq. An unambiguous assignment of the spectra was possible only for the compounds **2** and **3**. In the case of complexes **1**, **4** and **13** most of the signals were overlapped and therefore they could not be unambiguously assigned.

Table 3. $^{1}$H NMR Spectra of Rhodium(II) Complexes.

| Complex | δ, ppm, (J, Hz) |
|---|---|
| **1** in $CD_3OD$ | 3.334s($CH_3$), 4.598s($CHPh^{nc}$), 4.977s($CHPh^{c}$)·, 6.50-8.46m(Ph+diMe-phen) |
| **2** in $D_2O$ | 5.627s($CH^{c}$), 7.199dd($H^{8}$, $J_{78}$ = 8.28 Hz, $J_{89}$ = 5.13 Hz), 7.25 - 7.32m($H^{3}$ + $Ph^{nc}$), 7.424d($H^{9}$), 7.507($H^{6}$, the AB spectrum, $J_{56}$ = 8.90 Hz), 7.550($H^{5}$,the AB spectrum), 7.61 - 7.67m($H^{\beta}$, $H^{\gamma}$, $Ph^{c}$), 7.73 - 7.77m($H^{\alpha}$,$Ph^{c}$), 7.843d($H^{2}$, $J_{23}$ = 5.13 Hz), 7.997d($H^{7}$), 8.134d($H^{4}$, $J_{34}$ = 8.13 Hz) |
| **3** in $D_2O$ | 5.591s($CH^{c}$), 7.175dd($H^{8}$, $J_{78}$ = 8.22 Hz, $J_{89}$ = 5.13 Hz, $J_{89}$ = 5.13 Hz), 7.255dd($H^{3}$, $J_{23}$ = 5.13 Hz, $J_{34}$ = 8.22 Hz), 7.402d($H^{9}$), 7.488($H^{6}$, the AB spectrum, $J_{56}$ = 8.95 Hz), 7.536($H^{5}$,the AB spectrum), 7.59 - 7.65m($H^{\beta}$, $H^{\gamma}$, Ph), 7.70 - 7.73m($H^{\alpha}$), 7.821d($H^{2}$, $J_{23}$ = 5.13 Hz), 7.976d($H^{7}$), 8.117d($H^{4}$) |
| **4** in $D_2O$ | 1.80s($CH_3COO^{nc}$), 2.43s ($CH_3COO^{c}$), 2.27s ($CH_3$), 2.51s($CH_3$), 2.55s($CH_3$), 2.60s($CH_3$), 6.75-8.60m(aromatic H) |
| **5** in $D_2O$ | 7.346t(4H, $H^{\beta}$-phenyl$^{c}$, $J_{\alpha\beta}$=7.2 Hz), 7.419m(4H, $H^{3}$,$H^{8}$+2H, $H^{\gamma}$-phenyl$^{c}$), 7.560t(4H, $H^{\beta}$-phenyl$^{nc}$, $J_{\alpha\beta}$=7.6 Hz), 7.604s(4H, $H^{5}$, $H^{6}$, phen), 7.685t(2H, $H^{\gamma}$-phenyl$^{nc}$, $J_{\beta\gamma}$=7.4 Hz), 7.736d(4H, $H^{\alpha}$-phenyl$^{c}$), 8.153dd (4H, $H^{4}$, $H^{7}$, phen, $J_{34}$=8.2 Hz, $J_{24}$=0.8 Hz), 8.349d(4H, $H^{\alpha}$-phenyl$^{nc}$), 8.412d(4H, $H^{2}$, $H^{9}$, phen, $J_{23}$ = 5.1 Hz) |
| **6** in D2O | 1.592s($CH_3$), 1.616s($CH_3$), 1.677s($CH_3$), 1.704s($CH_3$), 1.784s($CH_3$), 1.799s($CH_3$), 6.99 - 7.15m(Ph), 7.22 - 7.35m(Ph) |
| **8** in $CD_3OD$ | 7.391($H^{5}$, $J_{56}$ = 5.5 Hz, $J_{45}$ = 7.2 Hz), 7.88 - 7.99m($H^{3}$ +$^{4}$), 8.355t(HCOO, $J_{RhH}$ = 4.2 Hz), 8.427d($H^{6}$) |
| **8** in $D_2O$ | 7.243m($H^{5}$), 7.74 - 7.82m($H^{3}$ + $H^{4}$), 8.232d($H^{6}$, $J_{56}$ = 5.2 Hz), 8.240t(HCOO, $J_{RhH}$ = 4.0 Hz) |
| **9** in $D_2O$ | 2.72s(3H, $CH_3$), 7.44ddd(4H, $H^{5}$ + $H^{5''}$, $J_{35}$ = 1.4 Hz, $J_{45}$ = 7.4 Hz, $J_{56}$ = 5.3 Hz), 7.83 - 8.03m(14H), 8.13d(4H, $H^{6}$ + $H^{6''}$) |
| **10** in $D_2O$ | 0.766t(6H, $CH_3{}^{nc}$, $^{3}J_{(HH)}$=7.40 Hz), 0.963t(6H, $CH_3{}^{c}$, $^{3}J_{(HH)}$=7.40 Hz), 1.428sxt(4H, $\mathit{CH_2}CH_3$, $^{3}J_{(HH)}$=7.40 Hz), 1.757sxt(4H, $\mathit{CH_2}CH_3{}^{c}$, $^{3}J_{(HH)}$= 7.40 Hz), 2.024t(4H, $\mathit{CH_2}$COO, $^{3}J_{(HH)}$=7.38 Hz), 2.676t (4H, $\mathit{CH_2}$COO, $^{3}J_{(HH)}$=7.38 Hz), 7.273ddd(4H, $H^{5}$,$H^{5'}$, $J_{56}$=5.6 Hz, $J_{45}$=7.5 Hz), 7.75-7.85m (8H, $H^{3}$, $H^{3'}$, $H^{4}$, $H^{4'}$), 8.147d(4H, $H^{6}$, $H^{6'}$) |
| **11** in $D_2O$ | 0.754t(6H, $CH_3{}^{nc}$, $^{3}J_{(HH)}$=7.40 Hz), 0.941t(6H, $CH_3{}^{c}$, $^{3}J_{(HH)}$=7.40 Hz), 1.161sxt(4H, $\mathit{CH_2}Me^{nc}$, $^{3}J_{(HH)}$=7.40 Hz), 1.388qnt(4H, $\mathit{CH_2}Et^{nc}$), 1.435sxt(4H, $\mathit{CH_2}Me^{c}$), 1.816qnt(4H, $CH_2Et^{c}$), 2.042t(4H, $\mathit{CH_2}COO^{nc}$), 7.490dd(4H, $H^{3}$, $H^{8}$, $J_{23}$=5.2 Hz), $J_{34}$=8.2 Hz), 7.588s(4H, $H^{5}$, $H^{6}$), 8.153d(4H, $H^{4}$, $H^{7}$), 8.342d(4H, $H^{2}$,$H^{9}$) |
| **12** in $D_2O$ | 0.765t(6H, $CH_3{}^{nc}$, $^{3}J_{(HH)}$=7.35), 0.901t(6H, $CH_3{}^{c}$, $^{3}J_{(HH)}$=7.35 Hz), 1.173sxt(4H, $\mathit{CH_2}CH_3{}^{nc}$, $^{3}J_{(HH)}$=7.35 Hz), 1.372sxt(4H, $\mathit{CH_2}CH_3{}^{c}$, $^{3}J_{(HH)}$=7.35 Hz), 1.401qnt(4H, $\mathit{CH_2}Et^{nc}$, $^{3}J_{(HH)}$=7.35 Hz), 1.724qnt(4H, $\mathit{CH_2}Et^{c}$, $^{3}J_{(HH)}$=7.48 Hz), 2.052t(4H, $\mathit{CH_2}COO^{nc}$, $^{3}J_{(HH)}$=7.42 Hz), 2.702t(4H, $\mathit{CH_2}COO^{c}$, $^{3}J_{(HH)}$=7.45 Hz), 7.275ddd(4H, $H^{5}$,$H^{5'}$, $J_{56}$= Hz, $J_{45}$=7.3 Hz, $J_{36}$=1.8 Hz), 7.76-7.85m(8H, $H^{3}$, $H^{3'}$, $H^{4}$, $H^{4'}$), 8.141(4H, $H^{6}$, $H^{6'}$) |
| **13** in $D_2O$ | 5.50s($CHOH^{c}$), 6.97q($H^{5'}$, $J_{5'6'}$ = 5,6 Hz), 7.02q($H^{5}$, $J_{56}$ = 5.6 Hz), 7.25d($H^{6'}$), 7.32m($Ph^{nc}$), 7.54 - 7.76m($Ph^{c}$ + $H^{3'}$ + $H^{3}$ + $H^{4'}$ + $H^{4}$ + $H^{6}$) |
| **14** in $CD_3OD$<br>**14** in $CDCl_3$ | 0.728t(12H, $CH_3$, $^{3}J(HH)$ = 7.30 Hz), 1.436sxt(8H, $\mathit{CH_2}$Me), 2.030t(8H, $\mathit{CH_2}$COO)<br>0.770t(12H, $CH_3$, $^{3}J(HH)$ = 7.30 Hz), 1.528sxt(8H, $CH_2$Me), 2.262t(8H, $\mathit{CH_2}$COO) |
| **15** in $CD_3OD$<br>**15** in $CDCl_3$ | 0.793t(12H, $CH_3$, $^{3}J(HH)$ = 7.30 Hz), 1.130sxt(8H, $\mathit{CH_2}$Me), 1.394qnt (8H, $\mathit{CH_2}$Et), 2.053(8H, $\mathit{CH_2}$COO)<br>0.796t(12H, $CH_3$, $^{3}J(HH)$ = 7.30 Hz), 1.1743sxt(8H, $\mathit{CH_2}$Me), 1.489qnt(8H, $CH_2$Et), 2.288t(8H, $CH_2$COO) |
| **16** in $(CD_3)_2CO$ | 7.28t(8H, $H^{\beta}$, $J_{\alpha\beta}$ = $J_{\beta\gamma}$ = 7.5 Hz), 7.40t(4H, $H^{\gamma}$), 7.87d(8H, $H^{\alpha}$) |

nc - noncoordinating ion, c - coordinating ligand, qnt = quintet, sxt = sextet

The complexes **1-13** and the ligands (phen, dmp, bpy, terpy and dmpq) were tested *in vitro* for antibacterial activity against Gram-positive *Staphylococcus aureus* 209 P (Oxford) and Gram-negative *Escherichia coli* ROW strains (Table 4). The highest activity against Gram-positive bacteria axhibited complexes **2-6** and **10-12**. Compounds **1** and **7** are much less active against *S. aureus* and all complexes show rather low activity against *E. coli.* The complexes **2**, **4**, **5** and **10-12** are considerably more active than the appropriate nitrogen ligands. However, the activity of complex **1** is lower than that of dmp. The activity of **3** is almost the same as that for non-coordinated phen and lower than activity of **2.** The complex **3** in the solid state contains chloro ligands coordinated along the Rh - Rh axis, however in solutions in water and in alcohols the prevailing form is $[Rh_2Cl\{PhCH(OH)COO\}_2(phen)_2(H_2O)]^+$ [2]. This indicate that complexes containing aqua molecules coordinated along Rh - Rh axis are more active than those with at least one chloro ligand substituted for $H_2O$. This is also confirmed by low activity of complex **9** with two chloro ligands bound along the Rh - Rh axis [26]. In order to get more information about the nature of the inhibitory activity of the investigated compounds, we have tested the survival of *S. aureus* in YP medium containing different concentration of complexes **2**, **4**, **5** and **6**. Viable counts were determined after 4 and 24 h and the results are presented in Fig. 2. All of the tested compounds delayed the beginning of the exponential growth of bacteria and clear-cut decrease of viable count in overnight cultures was observed at concentrations near to MIC. Values of viable count lower than the inoculum size indicate that the compounds have bactericidal activity. Among the tested compounds the most active is complex **5**.

It is known that the bactericidal activity of some antibiotics and chemoterapeutic agents depend on the size of thepopulation of treated microorganisms. Therefore, we have determined the survival of *S. aureus* cultures over the population range 10 to $10^6$ cells/$cm^3$ in the presence of **5**. The results presented in Table 5 indicate that the bactericidal activity of this compound depends on inoculum size. The bacteria are killed at the lowest concentration of **5** ($6 \cdot 10^{-7}$ M) when the inoculum size is equal to or less than $10^4$ cells/$cm^3$, however, for the populations equal to or greater than $10^5$ cells/$cm^3$, the complex **5** is effective at the concentration $1.25 \cdot 10^{-6}$ M.

Table 4. Minimal inhibitory concentration of rhodium compounds for *E. coli* ROW and *S. aureus* 209P Oxford*

| Complex | Concentration [M] | Percent of c.f.u. *E. coli* | Percent of c.f.u. *S. aureus* |
|---|---|---|---|
| **Control** | 0 | 100 | 100 |
| **1** | $1\ 10^{-5}$ | 71.9 | 71.7 |
| | $1\ 10^{-6}$ | 100 | 100 |
| **2** | $1\ 10^{-4}$ | 0 | 0 |
| | $5\ 10^{-5}$ | 0 | 0 |
| | $1\ 10^{-5}$ | 92.2 | 0 |
| | $5 \cdot 10^{-6}$ | - | 0.02 |
| **3** | $1\ 10^{-4}$ | 0 | - |
| | $5\ 10^{-5}$ | 0 | - |
| | $1\ 10^{-5}$ | 90.0 | 24.0 |
| **4** | $1\ 10^{-4}$ | 0 | - |
| | $1\ 10^{-5}$ | 100 | 0 |
| **5** | $1\ 10^{-4}$ | 0 | - |
| | $1\ 10^{-5}$ | 100 | 0 |
| **6** | $1\ 10^{-4}$ | 59.2 | - |
| | $1\ 10^{-5}$ | 84.0 | 0 |
| **7** | $1\ 10^{-4}$ | 0 | - |
| | $1\ 10^{-5}$ | 100 | 87.0 |
| **8** | $1\ 10^{-4}$ | 0 | - |
| | $5\ 10^{-5}$ | 0 | - |
| | $1\ 10^{-5}$ | 28.8 | 86.5 |
| **9** | $1\ 10^{-4}$ | 0 | - |
| | $1\ 10^{-5}$ | 100 | 77.9 |
| **10** | $1\ 10^{-4}$ | 0 | 0 |
| | $1\ 10^{-5}$ | 100 | 0 |

| | | | |
|---|---|---|---|
| **11** | $1\ 10^{-4}$ | 0 | 0 |
| | $5\ 10^{-5}$ | 0 | - |
| | $2.5\ 10^{-5}$ | 1 | - |
| | $1 \cdot 10^{-5}$ | 100 | 0 |
| | $5 \cdot 10^{-6}$ | - | 0 |
| | $1 \cdot 10^{-6}$ | - | 100 |
| **12** | $1\ 10^{-4}$ | 0 | 0 |
| | $5\ 10^{-5}$ | 0 | - |
| | $2.5\ 10^{-5}$ | 0.6 | - |
| | $1 \cdot 10^{-5}$ | 100 | 0 |
| | $5 \cdot 10^{-6}$ | - | 0 |
| | $1 \cdot 10^{-6}$ | - | 100 |
| **13** | $1\ 10^{-4}$ | 0 | 0 |
| | $5\ 10^{-5}$ | 0 | - |
| | $1\ 10^{-5}$ | 100 | 33 |
| **dmp** | $10^{-4}$ | 52 | 0 |
| | $10^{-5}$ | - | 0 |
| **dmpq** | $10^{-4}$ | 60 | 33 |
| **phen** | $10^{-4}$ | 0 | 0 |
| | $10^{-5}$ | 37 | 21 |
| **terpy** | $10^{-4}$ | 60 | 29 |
| **bpy** | $10^{-4}$ | 74 | 34 |

*determined on nutrient agar plates supplemented with tested rhodium complexes
- not tested

Some of the antibacterial agents, e.g. penicillin, are active only against growing cells. Thefore, it was interesting to find out whether the investigated compounds are active only against growing or also resting cells. The dependence of the survival of *S. aureus* treated with compound **5** on its concentration and time is presented in Table 6. It is evident that the complex **5** shows a killing effect on cells unable to grow. Incubation in saline even without the rhodium complexes leads to a decrease in viable count of *S. aureus* but in the presence of the compound **5** the diminution of the percent of colony forming units is much more drastic and depends on concentration. Analogous investigations were also carried out for complex **4**.

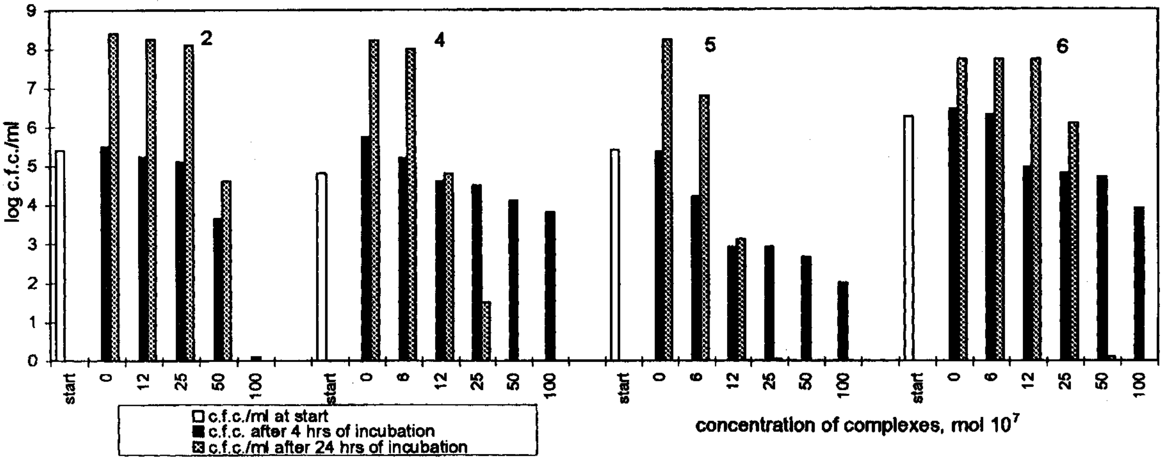

Fig. 2 Survival of S. Aureus P Oxford after 4 and 24 h exposition in YP broth treated with compounds **2**, **4**, **5** and **6** (c. f. c. - colony forming cells)

Table 5. The dependence of the activity of complex **5** on the inoculum size of *S. aureus* 209P Oxford in YP broth.

| Concentration [M] | Time [h] | Viable count/cm$^3$ | | | | | |
|---|---|---|---|---|---|---|---|
| 0 | 0 | $4.1\cdot10^6$ | $4.1\cdot10^5$ | $4.1\cdot10^4$ | $4.1\cdot10^3$ | $4.1\cdot10^2$ | $4.1\cdot10^1$ |
| $6\cdot10^{-7}$ | 4<br>24 | $5.3\cdot10^7$<br>$2.0\cdot10^8$ | $5.0\cdot10^5$<br>$3.6\cdot10^8$ | <10<br><10 | $1\cdot10^2$<br><10 | <10<br><10 | <10<br><10 |
| $1.25\cdot10^{-6}$ | 4<br>24 | $<1.0\cdot10^4<$<br>$10^4$ | $3.4\cdot10^5$<br>$<10^4$ | $1.2\cdot10^2$<br><10 | 20<br><10 | <10<br><10 | <10<br><10 |
| $2.5\cdot10^{-6}$ | 4<br>24 | $4.6\cdot10^5$<br>$<5\cdot10^5$ | $2.9\cdot10^3$<br>50 | 40<br><10 | <10<br><10 | <10<br><10 | <10<br><10 |
| $5\cdot10^{-6}$ | 4<br>24 | $3.8\cdot10^6$<br>$2.6\cdot10^5$ | $7.7\cdot10^2$<br><10 | <10<br><10 | <10<br><10 | <10<br><10 | <10<br><10 |

Table 6. Survival of *S. aureus* in saline treated with compound **5**.

| Concentration [M] | Time [h] | Percent of survival (No of colony units/cm$^3$) | |
|---|---|---|---|
| 0 | 0<br>2<br>4<br>24 | 100 ($13.5\cdot10^2$)<br>84<br>4.8<br><0.1 | 100 ($4.79\cdot10^4$)<br>79<br>8.4<br><0.1 |
| $9\cdot10^{-9}$ | 2<br>4<br>24 | 82<br>13.5<br><0.1 | 93.5<br>3.5<br><0.1 |
| $1.5\cdot10^{-7}$ | 2<br>4<br>24 | 79<br>4.9<br><0.1 | 83.3<br>3.5<br><0.1 |
| $1.25\cdot10^{-6}$ | 2<br>4<br>24 | 71<br><0.1<br>0 | 46<br><0.1<br>0 |
| $5\cdot10^{-6}$ | 2<br>4<br>24 | 30<br><0.1<br>0 | 19<br><0.1<br>0 |
| $1\cdot10^{-5}$ | 2<br>4<br>24 | 18<br><0.1<br>0 | 15<br><0.1<br>0 |

The results given in Table 7 indicate that compound **4** is also bactericide. This compound shows bactericidal activity against nongrowing cells suspended in saline (Table 8). The activity of the compound **5** was also tested against some of clinical *S. aureus* strains chosen at random. From among of 19 strains only 1 was resistant to the complex **5** in YP medium.

Table 7. Dependence of the survival of *S. aureus* on inoculum size and concentration of complex **4** in YP broth.

| Concentration [M] | Time [h] | Viable count/cm$^3$ | | | | | |
|---|---|---|---|---|---|---|---|
| 0 | 0<br>4<br>24 | $3.2\cdot10^6$<br>$4.5\cdot10^7$<br>$1.9\cdot10^8$ | $3.2\cdot10^5$<br>$9.2\cdot10^5$<br>$1.7\cdot10^8$ | $3.2\cdot10^4$<br>$4.5\cdot10^4$<br>$1.5\cdot10^8$ | $3.2\cdot10^3$<br>$9.1\cdot10^3$<br>$1.2\cdot10^8$ | $3.2\cdot10^2$<br>$2.9\cdot10^3$<br>$7.0\cdot10^7$ | 32<br>30<br>$4.1\cdot10^7$ |

| | | | | | | | |
|---|---|---|---|---|---|---|---|
| $6\cdot10^{-7}$ | 4 | $6.6\cdot10^{6}$ | $2.5\cdot10^{6}$ | $1.5\cdot10^{5}$ | $6.8\cdot10^{3}$ | $4.1\cdot10^{9}$ | $9.0\cdot10^{7}$ |
| | 24 | $2.5\cdot10^{8}$ | $2.0\cdot10^{8}$ | $1.5\cdot10^{8}$ | $8.6\cdot10^{7}$ | $5.1\cdot10^{7}$ | $4.6\cdot10^{7}$ |
| $1.25\cdot10^{-6}$ | 4 | $<1.0\cdot10^{6}$ | $8.7\cdot10^{5}$ | $3.2\cdot10^{4}$ | $4.4\cdot10^{3}$ | $3.6\cdot10^{2}$ | 85 |
| | 24 | $<1.0\cdot10^{6}$ | $<1.0\cdot10^{5}$ | $<1.0\cdot10^{5}$ | $1.1\cdot10^{4}$ | $5.2\cdot10^{3}$ | $1.5\cdot10^{3}$ |
| $2.5\cdot10^{-6}$ | 4 | $<1.0\cdot10^{6}$ | $5.3\cdot10^{5}$ | $6.5\cdot10^{3}$ | $3.9\cdot10^{3}$ | $2.9\cdot10^{2}$ | 60 |
| | 24 | <10 | <10 | <10 | <10 | <10 | 80 |
| $5\cdot10^{-6}$ | 4 | $<1.0\cdot10^{6}$ | $<2.7\cdot10^{4}$ | $5.5\cdot10^{2}$ | $3.2\cdot10^{2}$ | <10 | <10 |
| | 24 | <10 | <10 | <10 | <10 | <10 | <10 |

Table 8. Survival of *S. aureus* 209P Oxford in saline treated with compound **4**

| Concentration [M] | Time [h] | Viable count/cm$^3$ | |
|---|---|---|---|
| 0 | 0 | $1.0\cdot10^{7}$ | $1.0\cdot10^{6}$ |
| | 4 | $2.3\cdot10^{6}$ | $6.0\cdot10^{4}$ |
| | 24 | $8.2\cdot10^{4}$ | $4.2\cdot10^{2}$ |
| $6\cdot10^{-7}$ | 4 | $5.4\cdot10^{5}$ | $3.2\cdot10^{4}$ |
| | 24 | $3.3\cdot10^{4}$ | $3.8\cdot10^{1}$ |
| $1.25\cdot10^{-6}$ | 4 | $6.7\cdot10^{4}$ | $9.0\cdot10^{3}$ |
| | 24 | $3.9\cdot10^{2}$ | <10 |
| $2.5\cdot10^{-6}$ | 4 | $2.5\cdot10^{5}$ | $4.8\cdot10^{2}$ |
| | 24 | $1.8\cdot10^{2}$ | <10 |
| $5\cdot10^{-6}$ | 4 | $1.6\cdot10^{5}$ | <10 |
| | 24 | $2.4\cdot10^{1}$ | <10 |

The most active antibacterial agents against *S. aureus* are carboxylato complexes with ligands containing relatively bulky hydrocarbyl groups, i.e. $C_4H_9COO^-$, $C_6H_5COO^-$ and $C_6H_5CH(OH)COO^-$: $[Rh_2\{OOC(CH_2)_3CH_3\}_2(phen)_2(H_2O)_2]\{OOC(CH_2)_3CH_3\}_2$ (**11**) $[Rh_2\{OOC(CH_2)_3CH_3\}_2(bpy)_2(H_2O)_2]\{OOC(CH_2)_3CH_3\}_2$ (**12**), $[Rh_2(OOCC_6H_5)_2\text{-}(phen)_2(H_2O)_2](OOCC_6H_5)_2$ (**5**) and $[Rh_2\{OOCCH(OH)C_6H_5\}_2(phen)_2(H_2O)_2]\text{-}\{OOCCH(OH)C_6H_5\}_2$ (**2**). Activity of $[Rh_2\{OOCCH(OH)C_6H_5\}_2(bpy)_2(H_2O)_2]\text{-}\{OOCCH(OH)C_6H_5\}_2$ (**13**), however, is lower than that of complex **2**.

**Acknowledgements**
The authors are grateful to the KBN for support of this work (grant No. 2 P303 02005).

### References

[1] F.A. Cotton, R.A.Walton, *Multiple Bonds Between Metal Atoms,* Clarendon Press, Oxford, 1993; E.B. Boyar, S.D. Robinson, *Coord. Chem. Rev.*, **50**, 109(1983); T.R. Felthouse, *Progr. Inorg. Chem.*, **29**, 73(1982).

[2] F. Pruchnik, B.R. James, P. Kvintovics, *Can. J. Chem.*, **64**, 936(1986); F. Pruchnik, *Pure Appl. Chem.*, **61**, 795(1989); F. Pruchnik, M. Zuber, H. Pasternak, K. Wajda, *Spectrochim. Acta*, **34A**, 1111(1978); F.P. Pruchnik, J. Hanuza, K. Hermanowicz, K. Wajda-Hermanowicz, M. Zuber, *ibid.*; **45A**, 835(1989); H. Pasternak, F. Pruchnik, *Inorg. Nucl. Chem. Letters*, **12**, 591(1976); F. Pruchnik, M. Zuber, *Rocz. Chem.*, **51**, 1813(1977); F. Pruchnik, A. Jezierski, E. Kalecińska, *Polyhedron*, **10**, 2551(1991); T. Glowiak, F.P. Pruchnik , M. Zuber, *Polish J. Chem.*, **65**, 1749(1991); A. Szymaszek, F.P. Pruchnik, *Polish J. Chem.*, **66**, 1859(1992).

[3] J. Halpern, E. Kimura, J. Molin-Case, C.S. Wong, *J. Chem. Soc., Chem. Commun.*, 1207(1971).

[4] H. Pasternak, E. Lancman, F. Pruchnik, *J. Mol. Catal.*, **29**, 13(1985); H. Pasternak, F. Pruchnik, K. Wajda-Hermanowicz, M. Zuber, *Polish J. Chem.*, **63**, 619(1986); H. Pasternak, F.P. Pruchnik, *ibid.*, **66**, 865(1992).

[5] J.L. Bear, H.B. Gray Jr., L. Rainen, I.M. Chang, R. Howard, G. Serio, A.P. Kimball, *Cancer Chemother. Rep.* **59**, 611(1975); R.A. Howard, A.P. Kimball, J.L. Bear, *Cancer Res.*, **39**, 2568(1979).

[6] K.R. Dunbar, J.H. Matonic, V.P. Saharan, C.A. Crawford, G. Christou, *J. Am. Chem. Soc.*, **116**, 2201(1994).

[7] R.G. Hughes, J.L. Bear, A.P. Kimball, *Proc. Am. Assoc. Cancer Res.*, **13**, 120(1972).
[8] P.N. Rao, M.L. Smith, S. Pathak, R.A. Howard, J.L. Bear, *J. Nat. Cancer Inst.*, **64**, 905(1980).
[9] L.M. Hall, R.J. Speer, H.J. Ridgway, *J. Clin. Hematol. Oncol.*, **10**, 25(1980).
[10] E. Tselepi-Kalouli, N. Katsaros, *J. Inorg. Biochem.*, **40**, 95(1990).
[11] L.D. Dale, T.M. Dyson, D.A. Tocher, D.I. Edwards, *Anticancer Drug Res.*, **4**, 295(1989).
[12] S. Zyngier, E. Kimura, R. Najjar, *Braz. J. Med. Biol. Res.*, **22**, 397(1989).
[13] E.M. Reibscheid, S. Zyngier, D.A. Maria, R.J. Mistrone, R.D. Sinnistera, L.G. Couto, R. Najjar, *Braz. J. Med. Biol. Res.*, **27**, 91(1994).
[14] M.S. Nothenberg, G.K. Takeda, J. Najjar, *J.Inorg. Biochem.*, **42**, 217(1991).
[15] L. Trynda, F.P. Pruchnik, *J. Inorg. Biochem.*, **58**, 69(1995).
[16] R.C. Richmond, N.P. Farrel, H.K. Mathani, *Radiation Res.*, **120**, 403(1989); R.C. Richmond, H.K. Mathani, *Radiation Res.*, **127**, 36(1991).
[17] R. Chibber, I.J. Stratford, P. O'Neil, P.W. Sheldon, I. Ahmed, R. Lee, *Int. J. Radiation-Biol.*, **84** 513(1985).
[18] F.P. Pruchnik, D. Duœ, *J. Inorg. Biochem.*, **61**, 55(1996); F. Pruchnik, D. Duœ, *II Symposium on Inorganic Biochemistry and Molecular Biophysics, Proceedings* p.281, Wroclaw 1989.
[19] F.P. Pruchnik, G. Kluczewska, A. Wilczok, U. Mazurek, T. Wilczok, *J. Inorg. Biochem.*, in press, A. Wilczok, *Biological activity of Rhodium complexes in synchronous culture of Chlorella vulgaris.* Ph. D. Thesis, Silesian Academy of Medicine, Katowice 1990.
[20] R. J. Bromfield, R. H. Dainty, R. D. Gillard, B. T. Heaton, *Nature*, **223,** 735(1969).
[21] R. D. Gillard, Kem. *Kozlemenyek*, **47**, 107(1977).
[22] N. Farrel, *Transition Metal Complexes as Drugs and Chemotherapeutic Agents*, Kluwer, Dordrecht, 1989.
[23] F. Wanka, H.F.P. Joosten, W. J.De Grip, *Arch. Microbiol.*, **75**, 25(1970).
[24] G.A. Rempel, P. Legzdins, H. Smith, G. Wilkinson, *Inorg. Synth.*, **13**, 90(1972).
[25] C.R. Wilson, H. Taube, *Inorg. Chem.*, **14**, 405(1975).
[26] F.P. Pruchnik, F. Robert, Y. Jeannin, S. Jeannin, *Inorg. Chem.*, in press.
[27] Manual of clinical microbiology, eds. J. E. Blair, E. H. Lennette, J. P. Truant, *Am. Soc. Microbiol.*. Bethesda, 1970.
[28] L. Natkaniec, F.P. Pruchnik, *J. Chem. Soc., Dalton Trans.*, 3261(1994).
[29] H.Sigel, *Coord. Chem. Rev.*, 144(1995), 287.
[30] K. H. Scheller, H. Sigel, *J. Am. Chem. Soc.*, **105**, 5891(1983).
[31] O. Yamauchi, A. Odani, H. Masuda, H. Sigel, *Metal Ions Biol. Syst.*, **32**, 207(1996).

**Received: July 5, 1996 - Accepted: July 15, 1996 -**
**Received in revised camera-ready format: July 25, 1996**

